# Prevalence and pattern of cardiovascular magnetic resonance late gadolinium enhancement in highly trained endurance athletes

**DOI:** 10.1186/s12968-020-00660-w

**Published:** 2020-09-03

**Authors:** B. Domenech-Ximenos, M. Sanz-de la Garza, S. Prat-González, A. Sepúlveda-Martínez, F. Crispi, K. Duran-Fernandez, R. J. Perea, B. Bijnens, M. Sitges

**Affiliations:** 1 Radiology Department, Hospital Clinic, Barcelona, Spain; 2 Cardiovascular Institute, Institut d’Investigacions Biomèdiques August Pi I Sunyer (IDIBAPS), Barcelona, Spain; 3 Centro de Investigación Biomédica en Red Enfermedades Cardiovasculares (CIBERCV), Barcelona, Spain; 4 Barcelona Center for Maternal-Fetal and Neonatal Medicine Hospital Clínic and Hospital Sant Joan de Deu, Barcelona University, CIBER-ER, Barcelona, Spain; 5 Fetal Medicine Unit, Department of Obstetrics and Gynecology, Hospital Clínico - Universidad de Chile, Santiago de Chile, Chile; 6 Institut d’Investigacions Biomèdiques August Pi i Sunyer (IDIBAPS), Barcelona, Spain; 7 BCN Medtech, Universitat Pompeu Fabra, Barcelona, Spain; 8 ICREA, Barcelona, Spain

**Keywords:** Athletes, Fibrosis, Magnetic resonance imaging

## Abstract

**Background:**

Intensive endurance exercise may induce a broad spectrum of right ventricular (RV) adaptation/remodelling patterns. Late gadolinium enhancement (LGE) has also been described in cardiovascular magnetic resonance (CMR) of some endurance athletes and its clinical meaning remains controversial. Our aim was to characterize the features of contrast CMR and the observed patterns of the LGE distribution in a cohort of highly trained endurance athletes.

**Methods:**

Ninety-three highly trained endurance athletes (> 12 h training/week at least during the last 5 years; 36 ± 6 years old; 53% male) and 72 age and gender-matched controls underwent a resting contrast CMR. In a subgroup of 28 athletes, T1 mapping was also performed.

**Results:**

High endurance training load was associated with larger bi-ventricular and bi-atrial sizes and a slight reduction of biventricular ejection fraction, as compared to controls in both genders (*p* < 0.05). Focal LGE was significantly more prevalent in athletes than in healthy subjects (37.6% vs 2.8%; p < 0.001), with a typical pattern in the RV insertion points. In T1 mapping, those athletes who had focal LGE had higher extracellular volume (ECV) at the remote myocardium than those without (27 ± 2.2% vs 25.2 ± 2.1%; *p* < 0.05).

**Conclusions:**

Highly trained endurance athletes showed a ten-fold increase in the prevalence of focal LGE as compared to control subjects, always confined to the hinge points. Additionally, those athletes with focal LGE demonstrated globally higher myocardial ECV values. This matrix remodelling and potential presence of myocardial fibrosis may be another feature of the athlete’s heart, of which the clinical and prognostic significance remains to be determined.

## Key points

1. Highly trained endurance athletes showed a ten-fold increase in the prevalence of focal LGE as compared to control subjects, always confined to the hinge points.
2. Those athletes with focal LGE demonstrated globally higher myocardial ECV values.
3. This matrix remodelling and potential presence of myocardial fibrosis may be another feature of the athlete’s heart, of which the clinical and prognostic significance remains to be determined.

## Introduction

Long-lasting athletic training leads to structural, functional and electric cardiac adaptations known as the ‘athlete’s heart’ [1–3]. This occurs predominantly in endurance athletes since they undergo high training loads with a dynamic component. Nonetheless, adaptation to endurance exercise seems to be variable among individuals even when performing equivalent exercise training loads [4].

Late gadolinium enhancement (LGE) in the myocardium as assessed by contrast cardiovascular magnetic resonance (CMR) is associated with a poor prognosis in patients with cardiomyopathy [5–7]. Nevertheless, LGE has been also shown by CMR in some endurance athletes [8–12] and even in the general population [13–15]. However, the prevalence and prognostic relevance, of isolated LGE in athletes in the absence of overt structural cardiomyopathy, in terms of arrhythmogenesis, remains controversial [16].

CMR imaging is useful to assess cardiac morphology and function, and contrast enhanced CMR imaging with LGE correlates with histological indications of myocardial fibrosis in patients with previous myocardial infarction [17, 18]. In patients with an underlying cardiomyopathy, LGE has been suggested as a sensitive tool for the detection of focal myocardial fibrosis [5–7]. The presence and location of diffuse LGE and interstitial myocardial fibrosis, might be overlooked with conventional sequences. Therefore, the extracellular volume (ECV) assessment through T1-mapping sequences has been suggested as complementary information where ECV values have been correlated with myocardial fibrosis by histology [19–21] and, if increased, has been found to be an independent predictor of death and cardiac events in patients with heart failure [22].

The relationship between long-term endurance exercise and the presence of LGE is controversial. Current data are limited since a number of series that reported higher LGE in asymptomatic athletes [11, 23, 24], included older athletes with mixed patterns of LGE, such as subendocardial, transmural or subepicardial [11], or they only included male athletes, ignoring female competitive athletes [11, 23, 25].

Therefore, the purpose of this study was to evaluate the prevalence and the distribution pattern of LGE and ECV as potential indicators of myocardial fibrosis in highly-trained endurance athletes of both gender and to compare them to those observed in age- and gender-matched control subjects.

## Methods

### Study population

We recruited 93 triathletes from local triathlon clubs by invitation to participate in our study, between March 2015 and December 2017. Inclusion criteria were: (a) highly-trained endurance athletes (20–45 years) who had trained for a minimum of 12 h per week in the previous 5 years, (b) no cardiac symptoms or cardiovascular disease risk factors and (c) no family history of sudden cardiac death. The control group comprised 72 age- and gender-matched individuals. They were eligible if they only performed recreational sports of mild-moderate intensity for less than 3 h per week, and if they had no known history of cardiovascular disease.

The subjects were evaluated through the following tests: detailed anamnesis, physical examination, blood test and 12-lead surface electrocardiograms (ECG), and also a maximal cardiopulmonary exercise test in an upright cycloergometer (Ergoselected 100, Ergoline, Bitz, Germany) following an incremental ramp protocol. Study exclusion criteria were the presence of heart disease, renal failure (creatinine > 1,5 mg/dL), claustrophobia or any other contraindication for CMR. Lifetime training history and current training load were registered by direct reporting of the athletes. The study protocol complied with the declaration of Helsinki, institutional review board and ethics committee approval were obtained for this study. All subjects provided written informed consent.

### CMR imaging acquisition protocol

All athletes underwent CMR imaging with either a 3 T (*n* = 65) or a 1.5 T (*n* = 28) CMR scanner (Magnetom Trio or MagnetomAera, respectively; Siemens Healthineers, Erlangen, Germany), using a dedicated cardiac coil and ECG gating. Control subjects were scanned with the same 3 T scanner. After standardized CMR planning [26], conventional balanced steady-state free-precession cine imaging in a short-axis (8 mm slice thickness, 2 mm gap), covering the left ventricle (LV) and right ventricle (RV) from above the atrioventricular groove to the apex, in 2- and 4- chamber view were acquired [27]. In addition, after 10–15 min of 0.15 mmol/Kg gadobutrol (Gadovist®, Bayer Healthcare, Berlin, Germany) intravenous bolus injection, LGE images were obtained with standard phase-sensitive inversion recovery sequence matching cine images. Finally, in a subgroup of 28 athletes, whose studies were performed in the 1.5 T scanner, T1-mapping was also added using a modified look-locker inversion recovery sequence (MOLLI 5–3-3) in short axis (basal, mid ventricular and apical) and 4-chamber view. They were acquired at baseline and 15 min after a bolus of gadobutrol to quantify the ECV. Blood samples were drawn at the same moment of the CMR study and the hematocrit was determined. The acquired images were digitally stored in DICOM format.

### CMR data analysis

CMR images analysis were performed with specialized software (Argus, Siemens Medical Solutions, Germany) and were blindly evaluated by two experienced investigators, who had more than twelve years of CMR clinical expertise. Cardiac function quantification was performed using the summation of discs method; LV endo- and epicardial borders and RV endocardial borders were manually traced to quantify volumes at end-diastole (EDV) and end-systole (ESV), as well as the LV mass. Data were normalized to the subject’s body surface area (BSA). LGE was identified in contrast sequences and was deemed significant if the LGE pattern and extent was visually identified by both clinicians at least in two consecutive images. Native T1 and post-contrast T1 values were measured in regions of interest drawn in the LV septum (midventricular) and in the LV cavity (blood pool) to quantify ECV [ECV = (1 - Hematocrit) x (ΔR1_myocardium_/ΔR1_blood_)] [28]. Areas of focal LGE were excluded in order to evaluate these parameters unbiased from the presence of LGE.

### Statistical analysis

Data were analysed with SPSS for Windows (V.19.0, Statistical Package for the Social Sciences, International Business Machines, Inc., Armonk, New York, USA). A Gaussian distribution of all continuous variables was confirmed using a Kolmogorov–Smirnov test and values were reported as mean ± standard deviation (SD). To compare qualitative variables, a chi-squared test was used. An unpaired Student t-test was used to compare differences between athletes with and without LGE. Intraclass correlation coefficient for absolute agreement was used to evaluate the concordance between CMR measures performed by the two independent observers. A *p*-value of less than 0.05 was considered to indicate statistical significance.

## Results

### Baseline population characteristics

Characteristics of the male and female triathletes and control subjects are shown in Table 1. All four groups presented a similar distribution of age. Female athletes had lower BSA than male athletes, and BSA was also lower in athletes as compared to controls (*p* < 0.05). As expected, athletes showed a lower heart rate and higher maximal oxygen consumption (VO_2max_) as compared with the control group. All controls had a normal ECG and athletes presented with exercise-induced adaptive ECG changes such as incomplete right bundle branch block (male vs female: 20.4% vs 18.2%; *p* = 0.78) and mild or moderate sinus bradycardia (male vs female: 61% vs 64%; *p* = 0.81). No ECG pathological abnormalities were found in controls or in the athlete’s population.

**Table 1:** Baseline characteristics of the athletes and control subjects

	Athletes (***n*** = 93)	Controls (***n*** = 72)	***p***-value
**Age, years**	35.7 ± 5.8	34 ± 3.6	**0.038**
**BSA, m**^**2**^	1.78 ± 0.18	1.85 ± 0.21	**0.014**
**Systolic blood pressure, mmHg**	114 ± 15.9	113.9 ± 12.9	0.613
**Diastolic blood pressure, mmHg**	74.7 ± 9.8	76.1 ± 10.1	0.617
**Peak exercise systolic blood pressure, mmHg**	184.8 ± 35.6	NA	–
**HR, bpm**	57.5 ± 8.4	65.6 ± 10	**0.001**
**VO**_**2**_ **max, ml/min/kg**	43.7 ± 7.6	28.9 ± 7.8	**0.032**
**Training load per week, METs x min**	7619 ± 2837	NA	–
**Training load, years**	13.7 ± 7.7	NA	–

Values are mean SD or n (%). Values in **bold** indicate significant differences between groups

*BSA* Body surface area, *HR* Heart rate, *VO*_*2max*_ Maximal oxygen consumption, *MET* Metabolic equivalent of task, *NA* Not applicable

### Left and right cavity remodelling

Table 2 depicts cardiac dimensions and function as well as the results of contrast CMR in the different groups. Athletes showed a slight reduction of biventricular ejection fraction and larger biventricular volumes, particularly in the RV, resulting in a higher RV EDV-to-LV EDV ratio in athletes as compared to controls subjects (0.97 ± 0.1 vs 0.89 ± 0.1; *p* < 0.001). Male athletes presented larger biventricular and right atrial cavities as compared to female athletes (*p* < 0.05). LV mass and bi-atrial size were also larger in athletes as compared to controls.

**Table 2:** Cardiovascular magnetic resonance parameters in male and female athletes and control subjects

	Male			Female			Athletes vs controls
	Athletes (***n*** = 49)	Controls (***n*** = 42)	***p***-value	Athletes (***n*** = 44)	Controls (***n*** = 30)	***p***-value	
**LVEF, %**	56 ± 5	60 ± 5	**0.004**	58 ± 5	61 ± 4	**0.003**	**0.001**
**LV mass index, g/m**^**2**^	66 ± 10	49 ± 10	**0.001**	55 ± 8	40 ± 8	**0.001**	**0.001**
**LVEDVI, ml/m**^**2**^	109 ± 13	90 ± 15	**0.001**	98 ± 15	81 ± 9	**0.001**	**0.001**
**LVESVI, ml/m**^**2**^	47 ± 8	36 ± 8	**0.001**	41 ± 8	31 ± 5	**0.001**	**0.001**
**LVSV, ml/m**^**2**^	61 ± 8	53 ± 9	**0.001**	57 ± 10	50 ± 7	**0.001**	**0.001**
**RVEF, %**	52 ± 6	54 ± 5	0.201	54 ± 4	57 ± 5	**0.013**	**0.034**
**RVEDVI, ml/m**^**2**^	110 ± 15	82 ± 17	**0.001**	92 ± 16	72 ± 11	**0.001**	**0.001**
**RVESVI, ml/m**^**2**^	53 ± 11	38 ± 9	**0.001**	42 ± 8	31 ± 7	**0.001**	**0.001**
**RVSVI, ml/m**^**2**^	58 ± 9	44 ± 10	**0.001**	50 ± 9	41 ± 6	**0.001**	**0.001**
**LAAI, cm**^**2**^**/m**^**2**^	14 ± 2	12 ± 2	**0.001**	14 ± 2	13 ± 2	0.349	**0.001**
**RAAI, cm**^**2**^**/m**^**2**^	13 ± 2	12 ± 2	**0.002**	11 ± 2	10 ± 2	0.489	**0.012**
LGE present*	17 (35)	2 (5)	**0.001**	18 (41)	0	**0.001**	**0.001**
**Pericardial effusion**	1 (2)	5 (12)	0.059	15 (34)	16 (53)	0.100	0.068

Values are mean SD or n (%). Values in **bold** indicate significant differences between groups

*LVEF* Left ventricular ejection fraction, *LVEDVI* Left ventricular end-diastolic volume index, *LVESVI* Left ventricular end-systolic volume index, *LVSVI* Left ventricular stroke volume index, *RVEF* Right ventricular ejection fraction, *RVEDVI* Right ventricular end-diastolic volume index, *RVESVI* Right ventricular end-systolic volume index, *RVSVI* Right ventricular stroke volume indexd, *LAAI* Left atrial area index, *RAAI* Right atrial area index, *LGE* Late gadolinium enhancement

*Two female athletes CMR study did not include late gadolinium enhancement sequence due to technical problems

### Prevalence, localization and pattern of LGE

**Fig. 1:**
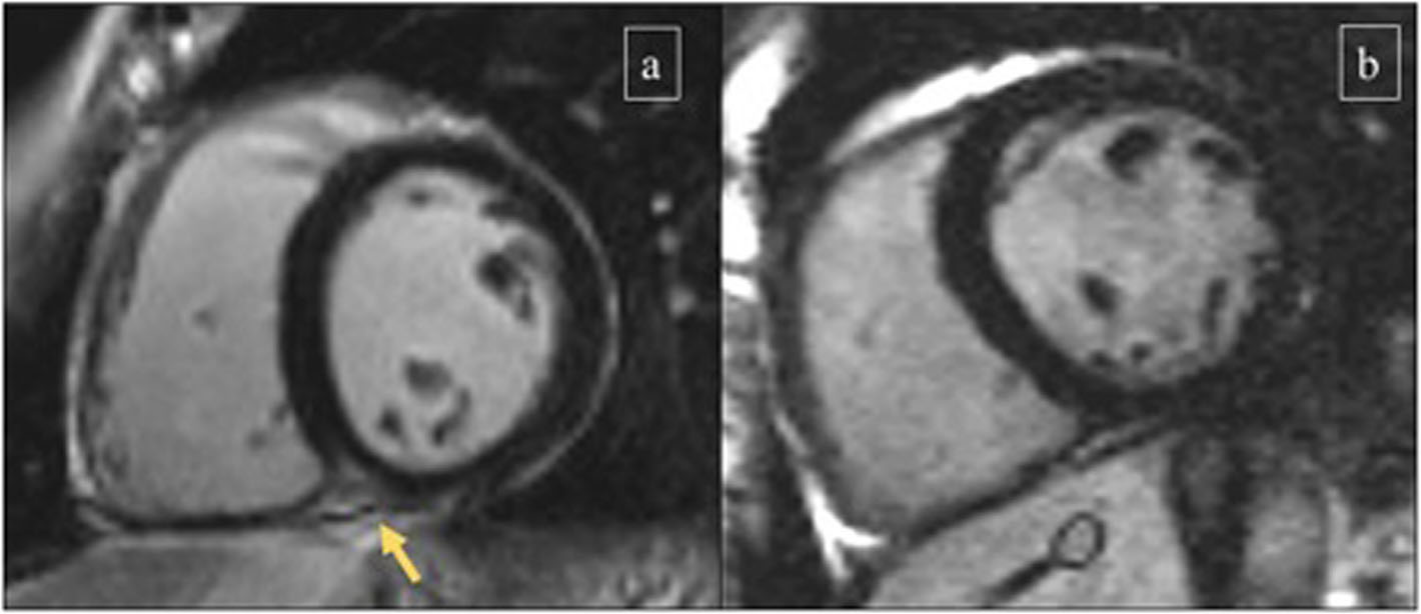
Late gadolinium enhancement (LGE) in two athletes. 1a, 34 year-old male triathlon athlete (training 15 h/week for 8 years), who had focal LGE confined where the right ventricular (RV) insertion into the inferior septum (arrow); compared to 1b, 35 year-old male triathlon athlete (training 14 h/week for 10 years), who presented with a normal study

**Fig. 2:**
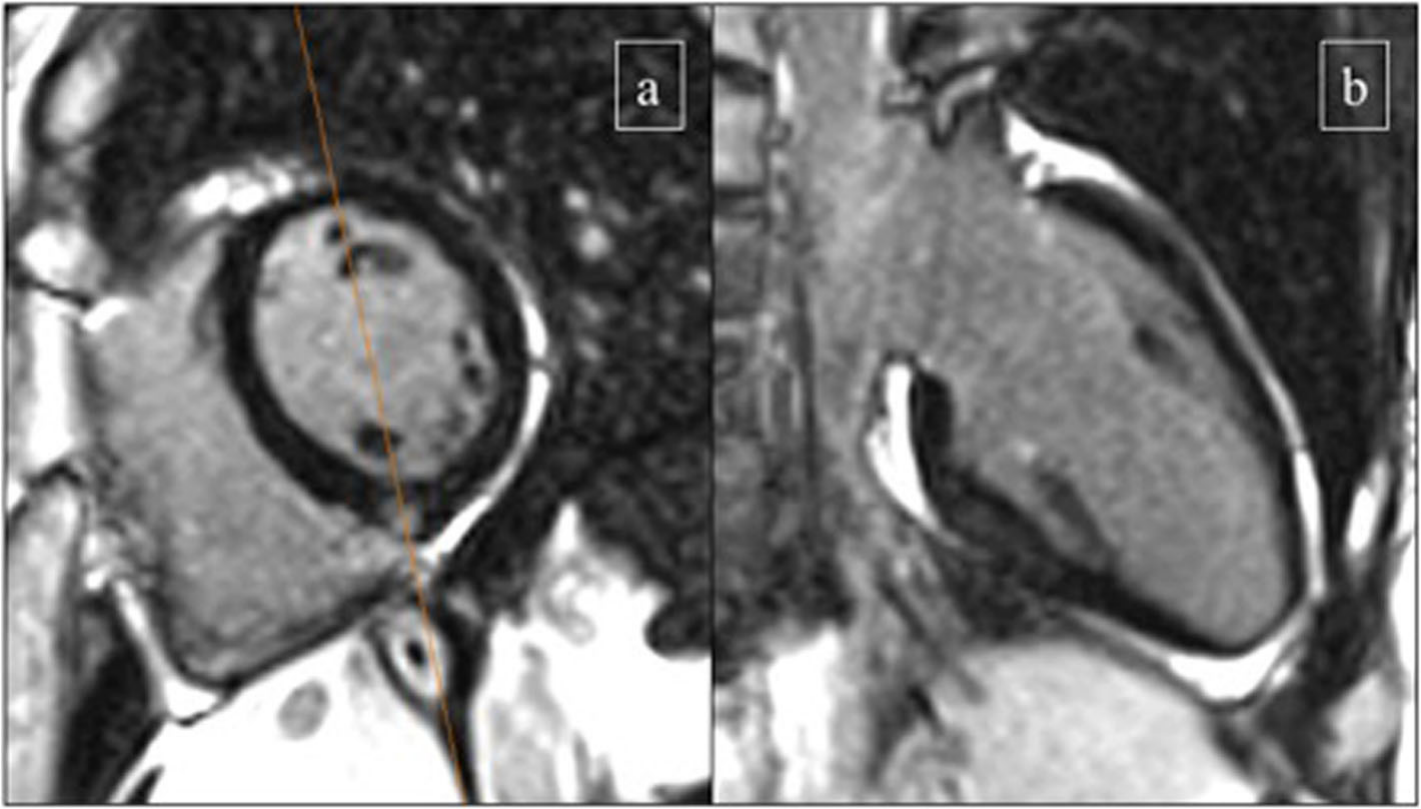
LGE in a 36 years old female triathlon athlete (training 13 h/week for 9 years), −who had focal LGE in the inferior RV insertion point; short axis (**a**) and long axis (**b**)

The presence of LGE was significantly more prevalent in athletes than in controls: 35 of 93 athletes (37.6%) showed LGE [17 of 49 males (34.7%) and 18 of 44 females (40.9%)], while only two of 72 (2.8%) controls had evidence of LGE. Thus, the presence of LGE was markedly increased in highly trained endurance athletes (in both genders) as compared to age- and gender-matched control subjects. All LGE^+^ triathletes had a similar pattern consisting of a small volume of focal LGE confined to the inferior interventricular septum, commonly where the RV attaches to the septum – insertion point or hinge point (Figs. 1 and 2). Other specific LGE patterns, such as subendocardial, transmural or subepicardial, were not identified in our population of healthy asymptomatic athletes.

### Differences between LGE^+^ and LGE^−^ athletes

Table 3 depicts population characteristics and CMR parameters for athletes with and without LGE. Both groups were of similar age, height, weight or BSA and had a similar training load.

**Table 3:** Clinical, athletic training and cardiovascular magnetic resonance parameters in male and female athletes with and without late gadolinium enhancement

	LGE^**+**^ Athletes (***n*** = 49)	LGE^**−**^ Athletes (***n*** = 44)	***p***-value
**Clinical parameters**			
Age, years	36 ± 6	35 ± 6	0.910
Female, n (%)	19 (39)	25 (57)	0.216
Weight, kg	68 ± 12	67 ± 11	0.643
Height, m	1.7 ± 0.9	1.7 ± 0.2	0.865
BSA, m^2^	1.8 ± 0.2	1.8 ± 0.1	0.569
Peak systolic blood pressure, mm Hg	188.5 ± 33.2	182.3 ± 38.1	0.995
HR, bpm	67.5 ± 11.3	59.9 ± 10.9	0.854
VO_2 max_, ml/min/kg	43.3 ± 8.2	44.0 ± 7.8	0.387
**Athletic training history**			
Active years, n	12 ± 5	12 ± 7	0.607
Training load per week (METs/h/min)	8856 ± 2959	7348 ± 2507	0.906
Endurance training during childhood^a^, n (%)	10 (20)	16 (36)	0.130
**CMR parameters**			
LVEF, %	57 ± 5	58 ± 5	0.372
LV mass indexed, g/m^2^	60 ± 12	61 ± 10	0.798
LVEDVI, ml/m^2^	102 ± 15	105 ± 14	0.381
LVESVI, ml/m^2^	43 ± 9	44 ± 8	0.792
LVSVI, ml/m^2^	57 ± 8	60 ± 9	0.125
RVEF, %	52 ± 4	54 ± 6	0.228
RVEDVI, ml/m^2^	101 ± 19	102 ± 18	0.717
RVESVI, ml/m^2^	48 ± 10	47 ± 11	0.909
RVSVI, ml/m^2^	52 ± 9	55 ± 10	0.257

Underlined values signify *p* < 0.05 by independent. Values are mean SD or n (%)

^a^Total number of subjects that performed high intense endurance training during childhood and the corresponding percentage within each group of the study population

The exercise-induced increase in systolic blood pressure was equivalent in LGE+ and LGE- athletes. Among men, their competition history revealed that those athletes with LGE^+^ had a trend toward more hours of training per week (14.9 ± 2.6 vs 13.3 ± 2.3; *p* = 0.06). Nevertheless, LGE^+^ athletes (both genders) showed no differences in LV and RV volumes and ejection fraction as compared to LGE^−^ athletes.

### T1 mapping and ECV

In a subgroup of 28 athletes (71% female), that were studied in the 1.5 T scanner, T1 mapping sequences were also included in the contrast CMR protocol. Globally, ECV mean values were 26.0 ± 2.3%, and within the range of previously reported normal intervals (ECV 25.3 ± 3.5%) [29, 30].

**Fig. 3:**
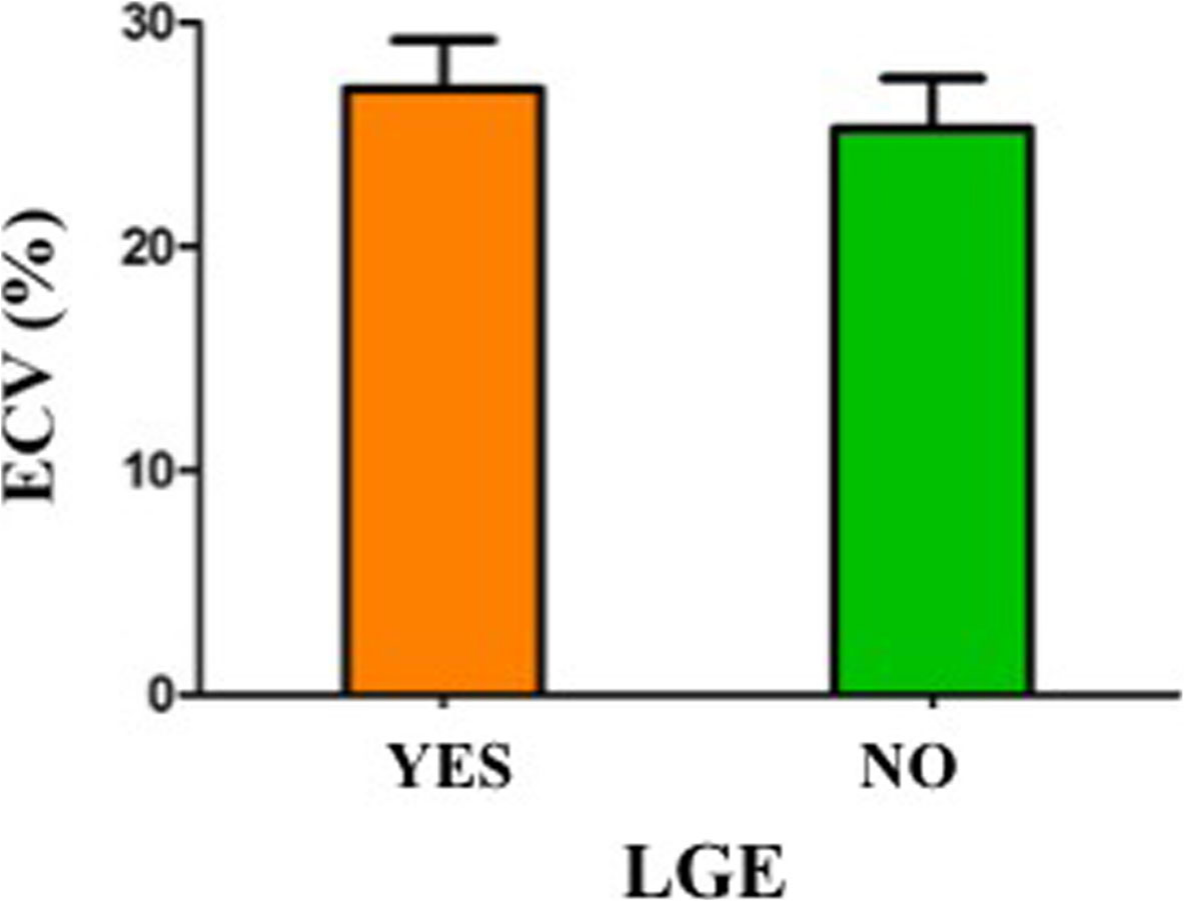
Values of extracellular volume fraction (ECV) according to the presence of late gadolinium enhancement (LGE). LGE+ athletes, had higher ECV at remote LV myocardium (areas where focal LGE was not identified) as compared to LGE- athletes, despite still within the reference limits of normality

Additionally, 13 of those 28 athletes (46.4%) had LGE confined to the interventricular septum. Those LGE+ athletes, had higher ECV at remote LV myocardium (areas where focal LGE was not identified) as compared to LGE- athletes (27.1% ± 2.2 vs 25.2% ± 2.1; *p* < 0.05), despite still within the reference limits of normality. These data are shown in Fig. 3. ECV above the reference limits of normality were only found in 2 of 28 athletes and they were not associated with gender (*p* = 0.82), training load (*p* = 0.59), or with structural (considering LV EDV; *p* = 0.32) or functional (considering left ventricular ejection fraction (LVEF); *p* = 0.11) ventricular changes. Concordance was high between the two observers for ECV values (intraclass correlation coefficient -absolute agreement- > 0.85).

## Discussion

The current study provides a comprehensive analysis of cardiac remodelling and the findings from CMR LGE and ECV assessment in asymptomatic highly trained endurance male and female athletes compared to age- and gender-matched controls.

The major findings of our study were: 1) as expected, highly trained endurance athletes showed an increase of biventricular and biatrial cavity sizes and a slight reduction of biventricular ejection fraction; 2) the presence of LGE, as a potential marker of focal fibrosis, was ten-fold more prevalent in athletes (both genders) as compared to controls, and it was always located in the inferior insertion point; 3) the presence of focal LGE did not result in significant differences in ventricular volumes and ejection fraction or in parameters of maximum aerobic capacity; and 4) athletes with LGE^+^ showed higher ECV values, despite they were globally still within the normal reference range.

### Left and right cavity remodelling

As expected, our results are in line with previous data proving that long-term endurance training promotes cardiac remodelling that consists of significant biventricular and biatrial dilatation in both genders and a slight reduction of biventricular ejection fraction (at rest). These changes are part of a common adaptation in trained athletes, known as the athlete’s heart [2, 3, 31, 32]. A mild decrease in resting LVEF and right ventricular ejection fraction (RVEF) was generally associated with more significant LV and RV enlargement; this is typically considered as adaptive since dilated ventricles have increased volume- and contractile reserve [31, 33].

### Prevalence and distribution of LGE on contrast CMR in athletes

Our study provides additional evidence that highly trained endurance athletes show a significantly higher prevalence of focal LGE than age- and gender-matched control subjects [4, 11, 23]. The prevalence of LGE, as a potential marker of focal fibrosis, was up to 37.6% in our group of athletes, ten-fold higher than that observed in the control group. This prevalence was significantly higher than reported in other series of athletes [11, 24, 34]. Even though Tahir et al. [34] did not find focal LGE in female athletes, we found a similar prevalence between male and female athletes (*p* > 0.05), as also previously described in other reports [24]; this could be due to the homogeneity of our cohort of athletes. Additionally, we did not find differences regarding training load between female and male athletes, unlike Tahir et al. [34], who noted that their female athletes reported less training load.

In our study, all athletes with LGE showed a similar pattern, consisting of a small volume of focal LGE confined to the interventricular septum, commonly where the RV attaches to the septum (the inferior insertion point or hinge point). This pattern corresponds to a typical non-ischemic substrate. Other specific LGE patterns, such as subendocardial, transmural or subepicardial, were not identified in our population.

This observation may be because our population was constituted by completely asymptomatic, otherwise healthy, young athletes, while in previous studies the athlete’s cohort was older [11, 12, 23, 34, 35] or had abnormal findings on their regular screening check-ups [36]. Breuckman and colleagues [11] studied a cohort of 102 veteran athletes and found that 12% had LGE, of which almost half showed a coronary artery disease pattern. Bohm et al. [12] in 33 endurance athletes found 3 of them having subepicardial LGE, which is typical for old myocarditis/pericarditis. Wilson and colleagues [23] in a small cohort of 12 veteran athletes found that 42% exhibited a non-coronary LGE pattern. They also included 17 young athletes in which no LGE was found. Likewise, Merghani et al. [35] included 152 master athletes, of which 14% revealed LGE. Nevertheless, they did not find relationship between myocardial fibrosis and exercise intensity, years of training, or number of competitions. Only in the study of Schnell et al. [36], athlete’s population was younger than ours. This study included 7 asymptomatic athletes who all showed LGE; including four with pathological T-wave inversions and two with ventricular arrhythmias on a screening exercise test. To our understanding, ours is the largest cohort of young, healthy and asymptomatic athletes published, and we assume that, unlike other studies, we probably did not find clearly pathological LGE patterns since our athletes were strictly recruited by invitation to participate in a research project, and not because they presented any type of clinical or electrical abnormality.

Regarding the presence of focal fibrosis in the insertion point, La Gerche et al. [32] has suggested that the RV may remodel slightly more than the LV in endurance athletes, since RV wall stress increases more than LV wall stress during exercise and this places an additional pressure load on the RV. Focal LGE in the RV inferior interventricular septum has been related to RV pressure overload and systolic pulmonary hypertension [37, 38]. Specifically, the presence of focal LGE in the RV inferior interventricular septum, as well as the extent of hyperenhancement, has been inversely related to measures of RV systolic function in patients with severe symptomatic pulmonary artery hypertension [37]. However, the clinical significance and the real impact on outcome of the presence of small regions of LGE in the RV insertion point, in asymptomatic subjects, are still uncertain and these aforementioned prognostic implications should not be extrapolated.

### Differences between LGE^+^ and LGE^−^ athletes

LGE^+^ athletes showed no significant differences in LV and RV volumes or function as compared to LGE^−^ athletes. However, other authors [11, 23, 24, 34] have reported that those athletes with LGE had been competing in endurance sports for longer and had greater RV EDV and lower RVEF. Additionally, our athletes did not show a hypertensive response to exercise and, unlike Tahir et al. [34], we did not find significant differences regarding the peak exercise systolic blood pressure between LGE+ and LGE- athletes.

Our data demonstrates balanced biventricular and biatrial dilatation with a slight reduction of biventricular ejection fraction in athletes with and without LGE; this may arise, given the inclusion criteria, from the fact that our athletes practise the same endurance sport discipline and have similar training load. Thus, all this data potentially suggests that this LGE pattern might be another feature of the athlete’s heart, related to local mechanical stress due to the exercise-induced cardiac overload. While LGE has been correlated to focal fibrosis in chronic infarction, LGE dynamics are still controversial and not fully understood in other settings. The presence of LGE indicates that the local matrix and fibre structure has changed, similarly to what happens indeed in histologic fibrosis and scar; however, LGE could also be present due to localized structural remodelling regardless of the presence of real fibrosis. Experimental models of endurance training might shed some light in the real clinical significance of the observed LGE in athletes.

### T1 mapping sequence and ECV assessment

Considering that the increased LV mass, observed in athletes, is due to an expansion of the cellular compartment, a decreased ECV is to be expected [39]. In fact, McDiarmid et al. presented data showing that increasing degrees of training load linearly increase myocyte hypertrophy and inversely ECV in the athletic group in a cohort of 30 athletes (athletes vs controls; 22.5% ± 2.6 versus 24.5% ± 2.2; *p* = 0.02) [39].

Nevertheless, in our study we did not find a decrease in ECV in athletes. And when we separately analysed those athletes with focal LGE confined to the inferior insertion point, we observed that they had slightly higher ECV at remote LV myocardium than those without LGE, despite still being within the limits of normality [29, 30]. These data are in line with previous published literature [34] suggesting that the potential myocardial fibrosis might involve the entire myocardium of athletes, with focal LGE, and would not be confined only to the LGE areas. There might be other explanations to the increase of ECV; Coelho-Filho and colleagues [40] worked with hypertensive patients with LV mass within the gender-specific normal range, and found that their ECV was significantly higher than in controls. They suggested that expansion of the ECV could precede significant increase of LV mass. Nevertheless, our patients did not have hypertension, which indeed was an exclusion criterion for the study, and there were no significant differences in LV mass between LGE+ and LGE- athletes.

Further studies with long-term follow-up are clearly warranted to understand if these slightly higher ECV values are another feature of the athlete’s heart or if this may be able to early identify athletes who are starting to potentially develop diffuse interstitial fibrosis and have indeed an abnormal adaptation to training.

### Study limitations

The quantification of the training load by assessing self-reported training load represents a potential limitation, because these parameters depend on the individual perception and accuracy of athletes. Currently, no long-term follow-up data on outcomes is available to assess the prognostic and clinical implications of the observed LGE. The T1 mapping sequence was available in only 30% of athletes, among whom 71% were female; thus, findings need to be confirmed in larger populations.

## Conclusions

Highly trained endurance athletes showed a ten-fold increase in the prevalence of focal LGE as compared to age-matched control subjects, always with a constant pattern confined to the hinge point of the inferior interventricular septum. This was observed together with balanced biventricular and biatrial dilatation. These findings suggest that this distribution pattern of LGE might be another feature of the athlete’s heart, with a clinical and prognostic significance that remains to be determined. In addition, those athletes with focal LGE demonstrated globally higher myocardial ECV values. The underlying mechanisms leading to these findings are unknown and beyond the target of the present study but might be related to local mechanical stress due to the exercise-induced cardiac (pressure) overload.

## Data Availability

All data generated or analysed during this study are included in this published article.

## Abbreviations

BSA: Body surface area
CMR: Cardiovascular magnetic resonance
ECG: Electrocardiogram
ECV: Extracellular volume fraction
EDV: End-diastolic volume
EDVI: End-diastolic volume index
ESV: End-systolic volume
ESVI: End-systolic volume index
HR: Heart rate
LAA: Left atrial area
LAAI: Left atrial area index
LGE: Late gadolinium enhancement
LV: Left ventricle/left ventricular
LVEF: Left ventricle ejection fraction
MOLLI Modified Look-Locker inversion recovery RAA: Right atrial area
RAAI: right atrial area index
RV: Right ventricle/right ventricular
RVEF: Right ventricle ejection fraction
VO2_max_: Maximal oxygen consumption
